# Sequential Organ Failure Assessment Score for Prediction of Mortality of Patients With Rhabdomyolysis Following Exertional Heatstroke: A Longitudinal Cohort Study in Southern China

**DOI:** 10.3389/fmed.2021.724319

**Published:** 2021-10-11

**Authors:** Ming Wu, Conglin Wang, Zheying Liu, Zhifeng Liu

**Affiliations:** ^1^Department of Critical Care Medicine and Hospital Infection Prevention and Control, Health Science Center, The Second People's Hospital of Shenzhen, First Affiliated Hospital of Shenzhen University, Shenzhen, China; ^2^Department of Critical Care Medicine, General Hospital of Southern Theatre Command of Peoples Liberation Army, Guangzhou, China; ^3^Key Laboratory of Hot Zone Trauma Care and Tissue Repair of Peoples Liberation Army, General Hospital of Southern Theatre Command of Peoples Liberation Army, Guangzhou, China

**Keywords:** exertional heatstroke, rhabdomyolysis, mortality, risk factors, SOFA score

## Abstract

**Background:** Heatstroke is a medical emergency that causes multi-organ injury and death without intervention, but limited data are available on the illness scores in predicting the outcomes of exertional heat stroke (EHS) with rhabdomyolysis (RM). The aim of our study was to investigate the Sequential Organ Failure Assessment (SOFA) score in predicting mortality of patients with RM after EHS.

**Methods:** A retrospective cohort study was performed, which included all patients with EHS admitted into the intensive care unit (ICU) of General Hospital of Southern Theater Command of Peoples Liberation Army from January 2008 to June 2019. RM was defined as creatine kinase (CK) > 1,000 U/L. Data, including the baseline data at admission, vital organ function indicators, and 90-day mortality, were reviewed.

**Results:** A total of 176 patients were enrolled; among them, 85 (48.3%) had RM. Patients with RM had a significantly higher SOFA score (4.0 vs. 3.0, *p* = 0.021), higher occurrence rates of disseminated intravascular coagulation (DIC) (53.1 vs. 18.3%, *p* < 0.001) and acute liver injury (ALI) (21.4 vs. 5.5%, *p* = 0.002) than patients with non-RM. RM was positively correlated with ALI and DIC, and the correlation coefficients were 0.236 and 0.365, respectively (both *p*-values <0.01). Multivariate logistics analysis showed that the SOFA score [odds ratio (OR) 1.7, 95% CI 1.1–2.6, *p* = 0.024] was the risk factor for 90-day mortality in patients with RM after EHS, with the area under the curve (AUC) 0.958 (95% CI 0.908–1.000, *p* < 0.001) and the optimal cutoff 7.5 points.

**Conclusions:** Patients with RM after EHS have severe clinical conditions, which are often accompanied by DIC or ALI. The SOFA score could predict the prognosis of patients with RM with EHS. Early treatment strategies based on decreasing the SOFA score at admission may be pivotal to reduce the 90-day mortality of patients with EHS.

## Introduction

Heatstroke is an acute medical emergency characterized by the central nervous system (CNS) dysfunction, multi-organ failure, and extreme hyperthermia (usually >40.5°C) with a mortality rate of 40 to 70% and a disability rate of 30%. It is typically classified as exertional heat stroke (EHS) and classical heatstroke (CHS) (1). Rhabdomyolysis (RM) is one of the complications in patients with EHS, which ranges from an asymptomatic illness with an elevated creatine kinase (CK) level to a life-threatening condition associated with extreme elevations in CK, electrolyte imbalances, acute kidney injury (AKI), or disseminated intravascular coagulation (DIC). However, the relationships between RM and organ function and prognosis are poorly documented.

Some studies have also shown that RM caused by heatstroke may be an important factor in initiating sepsis (2–6) and will further cause the disturbance of blood coagulation, which can easily lead to the occurrence of DIC (7) and AKI (8, 9). Research showed that the Sequential Organ Failure Assessment (SOFA) score was an independent risk factor affecting the survival of patients (10); therefore, treatments based on reducing the SOFA score may be pivotal for reducing the mortality of EHS complicated with AKI (11). However, so far there are few clinical studies on the clinical characteristics and prognosis of EHS complicated with RM, and there is no scoring system that can predict prognosis in patients with RM with EHS. To provide a reference for timely and effective treatment, a retrospective cohort study was designed in a tertiary-care teaching hospital in southern China over 10 years, in which the clinical characteristics, the relationships between RM and organ function, and risk factors and 90-day mortality were analyzed.

## Methods

### Study Design and Participants

This retrospective cohort study was performed in the intensive care unit (ICU) of General Hospital of Southern Theater Command of Peoples Liberation Army from January 2008 to June 2019. The inclusion criteria of EHS are as follows (1): patients exposed to high temperature, high humidity, and history of strenuous exercise, with an excessively high body temperature (central temperature higher than 40°C) or/and nervous system dysfunction (including delirium, cognitive impairment, coma, etc.). The exclusion criteria were as follows: (1) death or discharged within 24 h after admission, (2) incomplete data regarding key indicators, (3) incomplete outcome evaluation data obtained *via* telephone follow-up, and (4) a previous history of organ dysfunction, such as skeletal muscle disease and chronic kidney disease.

Comprehensive treatments were provided to all patients, such as body cooling, the volume of infusion, and anti-inflammation drugs. Meanwhile, organ function supports were provided for patients with RM under clinical guidelines if necessary, including appropriate hydration, alkalization of urine, blood purifications with polymer interception, and so on.

### Research Procedures

The basic characteristics of patients were reviewed, including the Acute Physiology and Chronic Health Evaluation II (APACHE II) score, SOFA score, Glasgow Coma Score (GCS), and inflammatory and organ function indicators at admission. The indicators included blood count (lymphocyte and platelets), kidney function markers [blood urea nitrogen (BUN) and serum creatinine (Scr)], liver function markers [total bilirubin, alanine aminotransferase (ALT), and aspartate aminotransferase (AST)], C-reactive protein (CRP), procalcitonin (PCT), cardiac markers [CK, MB isoenzyme of creatine kinase (CK-MB), MB, and cardiac troponin I (cTNI)], clotting factors [prothrombin time (PT), international normalized ratio (INR), activated partial thromboplastin time (APTT), thrombin time (TT), fibrinogen (FIB), and D-dimer], and blood transfusion during treatment. All patients were assigned to the RM group and the non-RM group according to the presence of RM. Survival time was defined as the duration from onset to death; when the survival time was longer than 90 days, it was recorded as 90 days. The main results, including the 90-day mortality, ICU time, and the total cost during hospitalization, were analyzed. The survival curve analysis was performed.

### Definitions

1. RM (12): General fatigue, muscle soreness, and soy sauce-like urine; elevated laboratory CK; and elevated non-cardiogenic MB. This study adopted the current consensus opinion that CK > 1,000 U/L or increased more than five times the normal level was considered as elevated CK, whereas an increase in CK due to cardiogenic shock (CK-MB/CK < 5%) was excluded.
2. DIC (13): International Society for Thrombosis and Haemostasis (ISTH) standard: An ISTH score ≥5 points.
3. AKI (14): The Kidney Disease: Improving Global Outcomes (KDIGO) standard: Scr increased to ≥26.5 μmol/L (≥0.3 mg/dl) within 48 h, Scr increased to ≥1.5 times the baseline within 7 days, or urine output <0.5 ml/(kg h) for 6 h.
4. Acute liver injury (ALI) (15): Plasma TBIL ≥ 34.2 μmol/L and INR ≥ 1.5, or with any grade of hepatic encephalopathy.
5. Lymphocytopenia (16): Absolute lymphocytes less than 0.8 × 10^9^/L.

### Statistical Analysis

The continuous variables conformed to a normal distribution are expressed as $\bar{x}\pm s.$ For continuous variables that did not conform to a normal distribution are presented as medians and interquartile ranges (IQRs), and the categorical data were summarized as numbers and percentages. Continuous variables were compared using the independent two-sample *t* test or Mann–Whitney *U* tests. Categorical variables were compared using the Chi-Square test or Fisher's exact test. Significant indicators were analyzed using single factor analysis. Indicators with a *P* value < 0.1 were included in the multivariate logistic regression (LR) model: OR (odds ratio) and 95% confidence interval levels (95% CI), and forward stepwise regression was used to gradually eliminate each variable. The predictive ability of SOFA score for 90-day mortality was assessed using the area under the receiver operating characteristic (AU-ROC) curve, and the optimal cutoff value was determined by Youden's index. We analyzed the 90-day mortality in the RM and non-RM groups using the Kaplan–Meier's method and assessed the differences by the log-rank test. The relationship between RM and organ injury was performed using the Pearson correlation analysis. Statistical analyses were performed using the IBM SPSS Windows version 23.0 (IBM Corp., Armonk, NY, USA), Empower (R) (http://www.empowerstats.com, X&Y solutions, Inc., Boston, MA, USA), and R (http://www.R-project.org) software. The *p-*values (two-tailed) less than 0.05 were considered statistically significant.

## Results

### Clinical Characteristics of the Patients With EHS

**Figure 1:**
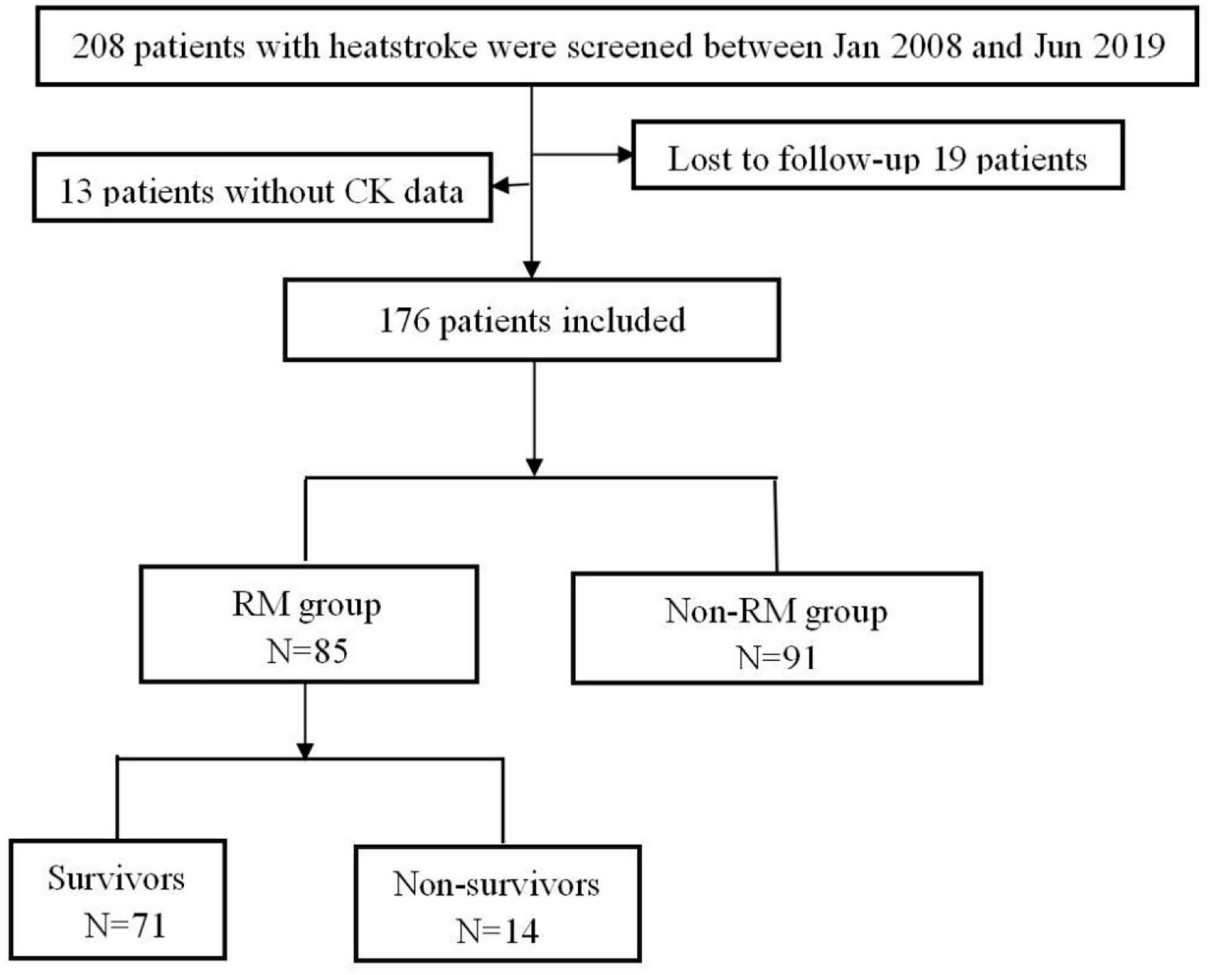
Flowchart of all excluded and included patients.

A total of 208 patients fulfilled the inclusion criteria; among them, 32 patients were excluded because of loss to follow-up or missing CK data. Finally, 176 patients were included, who were all men; among them, there were 91 patients (51.7%) without RM, and 85 patients (48.3%) with RM (Figure 1). There was no statistical difference in age between the two groups [20.0 vs. 22.0 (years), *p* = 0.472]. Compared with the patients with non-RM, the patients with RM had a significantly higher SOFA score (4.0 vs. 3.0, *p* = 0.021), higher incidence of DIC (53.1 vs. 18.3%, *p* < 0.001) and ALI (21.4% vs. 5.5%, *p* = 0.002). These patients also had lower lymphocyte and platelet, significantly increased CK, MB, PCT, PT, APTT, D-dimer, ALT, and AST, and higher blood transfusion proportion than patients with non-RM (all *p* < 0.05). However, there were no statistically significant differences in APACHE II (12.0 vs. 10.0, *p* = 0.285) and GCS scores (10.0 vs. 12.0, *p* = 0.429) and also in the incidence of lymphocytopenia (44.6 vs. 33.0%, *p* = 0.116) and AKI (48.8 vs. 39.6%, *p* = 0.218). Interestingly, the 90-day mortality of patients with RM was not significantly increased (16.5% vs. 8.8%, *p* = 0.124), whereas the total cost of hospitalization was particularly higher [51,986.3 vs. 31,810.5 (RMB), *p* = 0.036] than that of patients with non-RM (Table 1).

**Table 1:** Comparisons of clinical characteristics between patients with rhabdomyolysis (RM) and non-RM with exertional heat stroke (EHS).

**Variables**	**Non-RM (***n*** = 91)**	**RM (***n*** = 85)**	***P*** **-value**
APACHE II score, median (IQR)	10.0 (8.0–15.0)	12.0 (8.0–17.8)	0.285
SOFA score, median (IQR)	3.0 (2.0–5.0)	4.0 (2.0–9.0)	0.021
GCS score, median (IQR)	12.0 (7.0–14.0)	10.0 (7.0–13.0)	0.429
Age (years), median (IQR)	20.0 (19.0–26.5)	22.0 (19.0–27.0)	0.472
Lymphocyte (1 × 10^9^/L), median (IQR)	1.3 (0.7–2.3)	0.9 (0.5–1.7)	0.044
Platelets (1 × 10^9^/L), median (IQR)	186.0 (139.0–230.5)	106.0 (59.0–177.5)	<0.001
TBIL (μmol/L), median (IQR)	12.2 (8.6–21.5)	19.9 (13.3–37.6)	<0.001
ALT (U/L), median (IQR)	22.0 (15.0–41.5)	91.0 (33.0–868.0)	<0.001
AST (U/L), median (IQR)	36.0 (26.0–62.0)	173.0 (88.0–837.0)	<0.001
BUN (mmol/L), median (IQR)	5.5 (4.4–6.6)	6.2 (4.9–8.1)	0.022
Scr (μmol/L), median (IQR)	127.0 (94.0–150.5)	131.0 (93.0–186.0)	0.164
Cystatin C (mg/L), median (IQR)	1.0 (0.8–1.2)	1.0 (0.8–1.2)	0.838
CK (U/L), median (IQR)	372.0 (212.0–636.0)	2,918.0 (1,536.0–5,568.0)	<0.001
CK-MB (ng/ml), median (IQR)	26.0 (19.5–34.5)	72.0 (45.0–128.5)	<0.001
MB (ng/ml), median (IQR)	239.1 (73.3–646.5)	954.0 (317.8–1000.0)	<0.001
cTNI (pg/ml), median (IQR)	90.0 (26.4–281.1)	200.0 (30.0–699.9)	0.481
PT (s), median (IQR)	14.7 (13.7–16.4)	18.1 (15.8–26.2)	<0.001
INR, median (IQR)	1.2 (1.0–1.3)	1.5 (1.2–2.1)	0.003
APTT (s), median (IQR)	35.0 (31.6–40.9)	43.3 (37.4–76.5)	0.001
TT (s), median (IQR)	17.3 (16.3–18.5)	18.1 (16.8–29.8)	0.003
FIB (g/L), median (IQR)	2.5 (2.1–3.0)	2.4 (1.9–2.8)	0.111
D-Dimer (mg/L), median (IQR)	0.9 (0.4–3.7)	3.7 (0.9–10.1)	<0.001
CRP (mg/dl), median (IQR)	2.0 (0.4–3.7)	3.4 (3.2–7.2)	<0.001
PCT (ng/ml), median (IQR)	1.2 (0.6–2.4)	3.1 (1.4–5.3)	0.008
Transfusion, *N* (%)	14/90 (15.6%)	34/80 (42.5%)	<0.001
Lymphocytopenia, *N* (%)	30/91 (33.0%)	37/83 (44.6%)	0.116
DIC, *N* (%)	13/71 (18.3%)	34/64 (53.1%)	<0.001
AKI, *N* (%)	36/91 (39.6%)	41/84 (48.8%)	0.218
ALI, *N* (%)	5/91 (5.5%)	18/84 (21.4%)	0.002
90-day mortality, *N* (%)	8/91 (8.8%)	14/85 (16.5%)	0.124
ICU time (days), median (IQR)	4.0 (3.0–8.5)	6.0 (4.0–10.0)	0.024
Survival time (days), median (IQR)	90.0 (90.0–90.0)	90.0 (90.0–90.0)	0.118
Hospitalization costs (RMB), median (IQR)	31,810.5 (20,639.3–56,200.3)	51,986.3 (33,642.8–132,146.6)	0.036

*AKI, acute kidney injury; ALI, acute liver injury; APTT, activated partial thromboplastin time; APACHE II, Acute Physiology and Chronic Health Evaluation II; BUN, blood urea nitrogen; CK, creatine kinase; CK-MB, MB isoenzyme of creatine kinase; CRP, C-reactive protein; cTNI, cardiac troponin I; DIC, disseminated intravascular coagulation; FIB, fibrinogen; ICU, intensive care unit; INR, international normalized ratio; IQR, interquartile range; PCT, procalcitonin; PT, prothrombin time; Scr, serum creatinine; SOFA, Sequential Organ Failure Assessment; TT, thrombin time*.

### Comparisons of Survivors and Non-survivors With EHS Complicated With RM

Among the patients with RM induced by EHS, 71 survived (83.5%) and 14 died (16.5%). Non-survivors had higher APACHE II score [23.0 (21.0–23.5) vs. 11.0 (8.0–14.5), *p* < 0.001] and SOFA score at admission [12.0 (10.5–14.5) vs. 3.0 (2.0–6.0), *p* < 0.001], and lower GCS scores [6.0 (4.5–7.5) vs. 12.0 (8.0–13.5), *p* = 0.005]. In the non-survivor group, the organ function was worse than that in the survivor group, illustrated by the significantly increased total bilirubin, ALT, AST, Scr, Cystatin C, cTNI, and MB values (all *p* < 0.05), the worse blood coagulation (PT, INR, APTT, and D-dimer, all *p* < 0.001), and higher blood transfusion proportion (90 vs. 35.7%, *p* < 0.001). Furthermore, the non-survivors were easily complicated with DIC (100 vs. 42.3%, *p* < 0.001), AKI (100 vs. 38.6%, *p* < 0.001) and ALI (42.9 vs. 17.1%, *p* = 0.032) but there were no significant differences in the incidence of lymphocytopenia (61.5 vs. 41.4%, *p* = 0.180), inflammation index (PCT/CRP) (*p* > 0.05), and the length of ICU time [5.5 vs. 6.0 (days), *p* = 0.381] between survivors and non-survivors group. However, the total hospitalization costs in the non-survivor group were increased [156,820.3 vs. 45,182.6 (RMB), *p* < 0.001] (Table 2).

**Table 2:** Comparisons of clinical characteristics between survivors and non-survivors with RM induced by EHS.

**Variables**	**Survivor(***n*** = 71)**	**Non-survivor(***n*** = 14)**	***P*** **-value**
APACHE II score, median (IQR)	11.0 (8.0–14.5)	23.0 (21.0–23.5)	<0.001
SOFA score, median (IQR)	3.0 (2.0–6.0)	12.0 (10.5–14.5)	<0.001
GCS score, median (IQR)	12.0 (8.0–13.5)	6.0 (4.5–7.5)	0.005
Age (years), median (IQR)	22.0 (19.0–27.5)	21.5 (20.2–23.0)	0.311
Lymphocyte (1 × 10^9^/L), median (IQR)	1.0 (0.5–1.7)	0.5 (0.3–2.1)	0.368
Platelets (1 × 10^9^/L), median (IQR)	127.0 (72.8–186.2)	65.0 (29.0–84.0)	0.004
TBIL (μmol/L), median (IQR)	17.6 (12.9–33.5)	37.4 (21.2–103.6)	<0.001
ALT (U/L), median (IQR)	64.0 (31.0–648.2)	546.5 (95.0–1,648.2)	0.028
AST (U/L), median (IQR)	133.0 (77.5–708.0)	408.5 (306.5–1,849.2)	0.020
BUN (mmol/L), median (IQR)	5.8 (4.5–7.7)	7.9 (6.2–9.0)	0.597
Scr (μmol/L), median (IQR)	114.0 (88.5–149.0)	245.5 (210.0–283.0)	<0.001
Cystatin C (mg/L), median (IQR)	1.0 (0.8–1.2)	1.5 (1.1–2.8)	<0.001
CK (U/L), median (IQR)	2,486.0 (1,462.5–4,927.0)	6,196.0 (2,231.8–8,251.5)	0.583
CK–MB (ng/ml), median (IQR)	71.0 (44.0–105.0)	298.0 (98.0–374.0)	0.332
MB (ng/ml), median (IQR)	658.0 (228.0–1,000.0)	1,000.0 (1,000.0–1,000.0)	0.019
cTNI (pg/ml), median (IQR)	110.0 (20.0–343.1)	1,530.0 (1,019.0–3,860.0)	<0.001
PT (s), median (IQR)	17.1 (15.4–21.9)	38.6 (24.8–45.3)	<0.001
INR, median (IQR)	1.4 (1.2–1.9)	4.2 (2.8–5.0)	<0.001
APTT (s), median (IQR)	41.2 (36.4–49.8)	93.8 (68.5–123.8)	<0.001
TT (s), median (IQR)	17.7 (16.6–22.2)	40.7 (28.9–58.7)	<0.001
FIB (g/L), median (IQR)	2.5 (2.1–2.8)	1.3 (0.9–1.8)	0.002
D–Dimer (mg/L), median (IQR)	2.9 (0.7–6.6)	10.1 (10.0–20.0)	<0.001
CRP (mg/dl), median (IQR)	3.6 (3.2–7.7)	3.3 (3.3–3.3)	0.454
PCT (ng/ml), median (IQR)	3.0 (1.3–4.8)	4.6 (1.7–8.1)	0.433
Transfusion, *N* (%)	25/70 (35.7%)	9/10 (90%)	<0.001
Lymphocytopenia, *N* (%)	29/70 (41.4%)	8/13 (61.5%)	0.180
DIC, *N* (%)	22/52 (42.3%)	12/12 (100.0%)	<0.001
AKI, *N* (%)	27/70 (38.6%)	14/14(100.0%)	<0.001
ALI, *N* (%)	12/70 (17.1%)	6/14 (42.9%)	0.032
ICU time (days), median (IQR)	6.0 (4.0–10.8)	5.5 (5.0–8.8)	0.381
Survival time (days), median (IQR)	90.0 (90.0–90.0)	5.5 (5.0–8.8)	<0.001
Hospitalization costs (RMB), median (IQR)	45,182.6 (29,738.0–93,106.3)	156,820.3 (133,525.5–214,730.5)	<0.001

*AKI, acute kidney injury; ALI, acute liver injury; APTT, activated partial thromboplastin time; APACHE II, Acute Physiology and Chronic Health Evaluation II; BUN, blood urea nitrogen; CK, creatine kinase; CK-MB, MB isoenzyme of creatine kinase; CRP, C-reactive protein; cTNI, cardiac troponin I; DIC, disseminated intravascular coagulation; FIB, fibrinogen; ICU, intensive care unit; INR, international normalized ratio; IQR, interquartile range; PCT, procalcitonin; PT, prothrombin time; Scr, serum creatinine; SOFA, Sequential Organ Failure Assessment; TT, thrombin time*.

### The Relationship Between RM and Another Organ Injury in EHS

It was found by the Pearson correlation that RM was positively correlated with ALI and DIC, with the correlation coefficients of 0.236 and 0.365, respectively (both *p* < 0.01). However, it was not associated with AKI (*p* = 0.220) and lymphocytopenia (*p* = 0.117) when CK > 1,000 U/L was used as the serological diagnostic standard of RM (Table 3).

**Table 3:** The relationship between RM and another organ injury in EHS.

**Variables**	**RM**		
	**Correlation**	**95%CI**	***P*** **-value**
AKI	0.093	0.056, 0.238	0.220
ALI	0.236	0.090, 0.371	0.002
Lymphocytopenia	0.119	−0.030, 0.263	0.117
DIC	0.365	0.209, 0.503	<0.001

*AKI, acute kidney injury; ALI, acute liver injury; DIC, disseminated intravascular coagulation*.

### Risk Factors of 90-Day Mortality for EHS Complicated With RM

**Figure 2:**
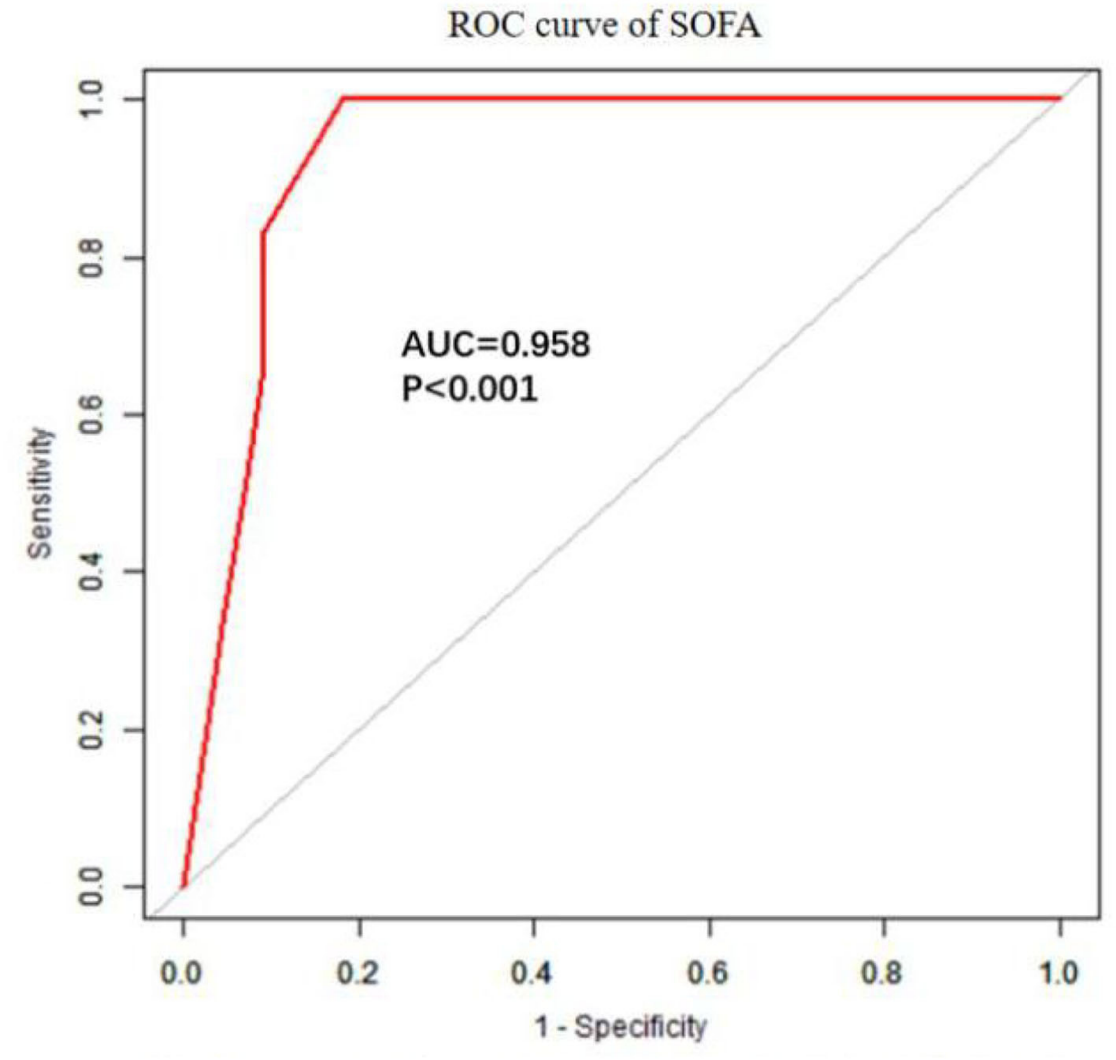
The ROC curve of SOFA in predicting 90-day mortality with patients with RM induced by EHS. EHS, exertional heat stroke; RM, rhabdomyolysis; ROC, receiver operating characteristic; SOFA, Sequential Organ Failure Assessment.

**Figure 3:**
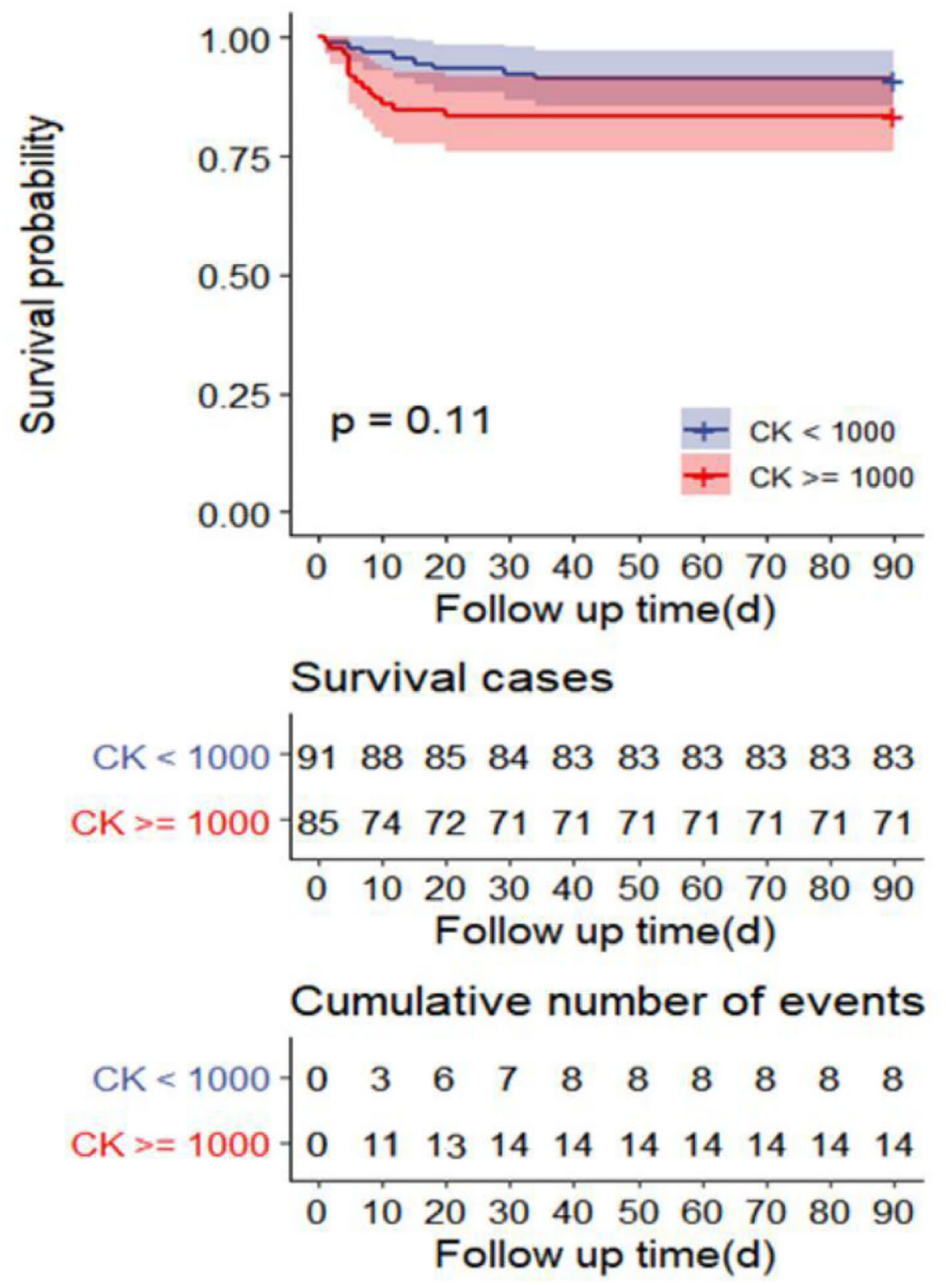
Survival curves of 90-day mortality rate in the R) group (CK ≥ 1,000 U/L) and non-RM group (CK < 1,000 U/L). CK, creatine kinase; RM, rhabdomyolysis.

The univariate analysis showed that APACHE II, SOFA, GCS, Cystatin C, MB ≥ 1,000 ng/ml, INR, FIB, and D-dimer were closely related to the 90-day mortality of patients with RM (all *p* < 0.001). The multivariate logistic regression showed that the SOFA score [OR 1.7 (1.1, 2.6), *p* = 0.024] was an independent risk factor affecting 90-day mortality in patients with EHS complicated with RM (Table 4). The area under the ROC curve for prediction of mortality based on the SOFA score was 0.958 (95% CI 0.908–1.000, *p* < 0.001), the optimal cutoff was 7.5 points, with SEN 100% and SPE 83.7% (Figure 2). However, there was no significant difference in the 90-day mortality between patients with RM and non-RM (*p* = 0.11) (Figure 3).

**Table 4:** Risk factors for 90-day mortality with RM induced by EHS.

**Variables**	**Univariate OR (95%CI)**	**Multivariate OR (95%CI)**
	***P*** **-value**	***P*** **-value**
APACHE II score	1.5 (1.3, 1.9) <0.001	1.3 (0.8, 1.9) 0.272
SOFA score	2.2 (1.5, 3.1) <0.001	1.7 (1.1, 2.6) 0.024
GCS score	0.6 (0.4, 0.8) <0.001	1.2 (0.7, 2.2) 0.497
DIC	18.4 (5.0, 67.2) <0.001	9.1 (0.2, 489.7) 0.277
AKI	19.7 (4.4, 87.5) <0.001	9.5 (0.1, 748.2) 0.312
Cystatin C	2.7 (1.6, 4.5) <0.001	NA
MB ≥ 1,000 ng/ml	7.4 (2.7, 20.4) <0.001	NA
INR	2.1 (1.4, 3.0) <0.001	NA
FIB	0.2 (0.1, 0.5) <0.001	NA
D-dimer	1.1 (1.0, 1.2) <0.001	NA
ALI	1,486,691.7 (0.0, Inf) 0.992	NA

*Adjust model adjust for: Age. AKI, acute kidney injury; ALI, acute liver injury; APACHE II, Acute Physiology and Chronic Health Evaluation II; DIC, disseminated intravascular coagulation; FIB, fibrinogen; GCS, Glasgow Coma Score; OR, odds ratio; SOFA, Sequential Organ Failure Assessment*.

## Discussion

In this study, we observed clinical characteristics and risk factors in critically ill patients with RM induced by EHS in southern China. When CK ≥ 1,000 U/L was used as the serological diagnostic standard of RM, the results showed that the SOFA score of patients with RM was higher than that of patients with non-RM, which were mainly involving ALI and DIC. While non-survivors with EHS complicated with RM had a higher incidence of DIC and AKI at admission. The SOFA score at admission was an independent risk factor for 90-day mortality in patients with RM following EHS.

There are many causes leading to RM. Patients with RM caused by EHS were often doing strenuous exercise under high temperature and humidity, which are different from crush syndrome. Vascular endothelial cells are more severely damaged due to the high temperature, which leads to a higher incidence of DIC. On the other hand, heatstroke could induce ALI *via* IL-1β and HMGB1-induced pyroptosis (17). It may be related to the pathogenesis as followed. RM releases myoglobin that can be decomposed into myosin, which plays an important role in the coagulation cascades, including both coagulation factors and fibrinolysis (18). In addition, some nuclear proteins are released by muscle cell injuries, such as histone 3 (19, 20) and HMGB1 (21), which can activate platelets and then lead to the occurrence and development of DIC and ALI. Previous studies have found that the renal tubular injury in RM caused by crush syndrome is mainly caused by apoptosis (22), which shows that RM is prone to AKI. The mechanism of its occurrence may include the following two aspects or the result of a combination: the deficiency of effective circulating blood volume caused by fluid loss and dehydration (23), and the mechanical obstruction of renal tubules caused by MB released RM (24). It has even been reported that myoglobin oxidative stress directly leads to renal tubular epithelial injury, but the specific mechanism of this injury is not completely clear yet (25). Furthermore, our study also showed that non-survived patients with RM were more likely complicated with AKI and DIC (*p* < 0.001). The reasons may be caused by the direct damage to vascular endothelial cells due to heat shock (26) and significantly reduced renal perfusion due to DIC, which are different from crush syndrome. Therefore, it is necessary to further explore the mechanism of RM on renal tubules under heatstroke.

Due to the imbalance between production and dissipation of heat, the occurrence of EHS is high when doing strenuous exercise under high temperature conditions. It often damages from CNS, striated muscle, kidney, and the coagulation system. The mechanism may be related to vascular endothelial cell damage, and the activation of inflammatory cells and platelets (21). A single-center retrospective study of 140 critical patients with severe heatstroke found that RM at admission was an independent risk factor for mortality (27). However, there was no further study on the effects of RM and other key organ functions and mortality. Therefore, we analyzed the effect of RM on the indicators of key organ functions and 90-day mortality by using RM as an exposure factor. The results showed that patients with EHS complicated with RM had more severe organ injuries. RM was positively correlated with ALI and DIC (all *p* < 0.01*)* whereas not associated with AKI (*p* = 0.220) and lymphocytopenia (*p* = 0.117), but there was no difference in 90-day mortality (*p* = 0.11) between patients with RM and non-RM. We speculated that the underlying reason is the inaccuracy of CK ≥ 1,000 U/L as the serological diagnostic criteria for RM in evaluating RM and organ function. In addition, there were other factors leading to AKI, such as glomerular perfusion pressure decreased because of the lower cardiac output, renal tubular apoptosis, renal interstitial edema, inflammatory exudation, and so on (25, 28). Multicenter prospective studies are needed to confirm CK thresholds at different organ injuries.

Multivariate logistic regression showed that only the SOFA score was an independent risk factor for 90-day mortality in patients with EHS complicated with RM, but not the APACHE II score. The APACHE II score is an important scoring system for evaluating the prognosis of critically ill patients, which involves age and chronic health. However, our patients were previously healthy and had a median age of 21 years. In addition, because the APACHE II score excluded some vital acute organ functions including coagulation function and liver function, it is not as comprehensive as that of the SOFA score. Therefore, the APACHE II score is not appropriate to evaluate prognosis in young patients with EHS. The optimal cutoff for the prediction of 90-day mortality based on the SOFA score was 7.5 points, with SEN 100% and SPE 83.7%. Moreover, SOFA scores in survivors and non-survivors with RM induced by EHS were 3.0 and 12.0, respectively. This indicates that the SOFA score can accurately predict the 90-day mortality of patients with RM induced by EHS. Survival curves showed there was no significant difference in the 90-day mortality between patients with RM (CK ≥ 1,000 U/L) and non-RM. Because the serologic diagnostic standard of RM with CK ≥ 1,000 U/L is too lenient, it does not reflect the true organ function status and predict the prognosis of patients. Multicenter prospective studies are needed to confirm CK thresholds for 90-day mortality in different disease states.

There are many measures that can be used to treat RM in ICU, including removal of MB by blood purification (29), antioxidation (30), anti-inflammation (31), and so on. Only the SOFA score was an independent risk factor for mortality, suggesting that the follow-up treatment with the primary aim of protecting key organ function is an important way to reduce mortality.

This study has some limitations. It was a single-center retrospective cohort study with a comparatively small number of cases. In addition, this study excluded 32 patients, which may cause selective bias in the results. Because all the patients were male and the average age was relatively young, though the type of heatstroke was restricted to EHS, the results do not fully reflect the overall conditions of the heatstroke population. Expanding the sample size and employing a prospective cohort study should be designed to achieve higher-level clinical results in the subsequent studies.

## Conclusions

Patients with RM after EHS have severe clinical conditions, which are often accompanied by DIC or ALI. The SOFA score was an important independent risk factor for 90-day mortality in patients with EHS complicated with RM. Early treatment strategies based on decreasing the SOFA score at admission may be pivotal to reduce the 90-day mortality rate of patients with EHS.

## Data Availability Statement

The original contributions presented in the study are included in the article/supplementary material, further inquiries can be directed to the corresponding author/s.

## Ethics Statement

The studies involving human participants were reviewed and approved by the Research Ethics Committee of the General Hospital of Southern Theatre Command of Peoples Liberation Army (HE-2020-09). Written informed consent for participation was not required for this study in accordance with the national legislation and the institutional requirements.

## Author Contributions

ZFL and MW contributed to the study concept and design. MW, CW, and ZYL collected the data. MW and CW performed the statistical analysis. ZFL, MW, and CW drafted the manuscript. All the authors had full access to all the data in the study and take responsibility for the integrity of the data and the accuracy of the data analysis.

## Funding

This study was supported by grants from the National Natural Science Foundation of China (No. 82072143), Natural Science Foundation of Guangdong Province of China (No. 2021A1515010170), the Peoples Liberation Army Logistics Research Project of China (18CXZ030 and BLJ20J006), Sanming Project of Medicine in Shenzhen (SZSM20162011), Shenzhen Science and Technology Innovation Commission (JCYJ20170306091335008 and JCYJ20190806163603504), and Clinical Research Project of Shenzhen Municipal Health Commission (SZLY2017007).

## Conflict of Interest

The authors declare that the research was conducted in the absence of any commercial or financial relationships that could be construed as a potential conflict of interest.

## Publisher's Note

All claims expressed in this article are solely those of the authors and do not necessarily represent those of their affiliated organizations, or those of the publisher, the editors and the reviewers. Any product that may be evaluated in this article, or claim that may be made by its manufacturer, is not guaranteed or endorsed by the publisher.
